# Queries on the Influence of Tobacco

**Published:** 1850-10

**Authors:** 


### QUERIES ON THE INFLUENCE OF TOBACCO.

We have been requested by a much respected friend, to lay before our readers, some queries recently addressed to him by an English Surgeon in relation to the influence of Tobacco upon the health, in hopes that some of our readers would be able to afford the desired information. He says, " many circumstances of late have occurred, in which I have seen the most injurious effects of the use of tobacco upon the nervous, circulatory, and digestive functions. A friend of mine became a perfect hypochondriac by the use of snuff, and was at once relieved by leaving it off. Upon returning to the use of it, he again suffered as before, and was again relieved by ceasing to take it. Here there was no doubt. Another gentleman was covered with an eruption resembling psoriasis, from head to foot, and got well immediately when he left off the use of snuff. Three times he suffered a relapse upon taking snuff, and was cured by leaving it off. In many smokers, I may say all, I have found heart disease or confirmed dyspepsia. If you can help me with any statistic accounts of disease of the heart and arteries, of brain and nervous system, and of the stomach and chylopoietic viscera, and cancer of the mouth and lips, I should feel greatly obliged. If to these you could add any data of the use of tobacco by the sufferers, it would greatly enhance their value."

The subject is a deeply interesting one, and we trust that out of the large experience of our readers, something may be gained to illustrate the effects of this agent upon the economy.

